# Quantum Probability and Randomness

**DOI:** 10.3390/e21010035

**Published:** 2019-01-07

**Authors:** Andrei Khrennikov, Karl Svozil

**Affiliations:** 1 International Center for Mathematical Modeling in Physics, Engineering, Economics, and Cognitive Science, Linnaeus University, 351 95 Växjö, Sweden; 2 Institute for Theoretical Physics, Vienna University of Technology, Wiedner Hauptstrasse 8-10/136, 1040 Vienna, Austria

**Keywords:** quantum foundations, probability, irreducible randomness, random number generators, quantum technology, entanglement, quantum-like models for social stochasticity, contextuality

The recent quantum information revolution has stimulated interest in the quantum foundations by perceiving and re-evaluating the theory from a novel information-theoretical viewpoint [1,2,3,4,5]. Quantum probability and randomness play the crucial role in foundations of quantum mechanics.

It might not be totally unreasonable to claim that, already starting from some of the earliest (in hindsight) indications of quanta in the 1902 Rutherford–Soddy exponential decay law and the small aberrations predicted by Schweidler [6], the tide of indeterminism [7,8] was rolling against chartered territories of fin de siécle mechanistic determinism. Riding the waves were researchers like Exner, who already in his 1908 inaugural lecture as *rector magnificus* [9] postulated that irreducible randomness is, and probability theory therefore needs to be, at the heart of all sciences; natural as well as social. Exner [10] was forgotten but cited in Schrödinger’s alike “Zürcher Antrittsvorlesung” of 1922 [11]. Not much later Born expressed his inclinations to give up determinism in the world of the atoms [12], thereby denying the existence of some inner properties of the quanta which condition a definite outcome for, say, the scattering after collisions.

Von Neumann [13] was among the first who emphasized this new feature which was very different from the “in principle knowable unknowns” grounded in epistemology alone. Quantum randomness was treated as *individual randomness*; that is, as if single electrons or photons are sometimes capable of behaving acausally and irreducibly randomly. Such randomness cannot be reduced to a variability of properties of systems in some ensemble. Therefore, quantum randomness is often considered as *irreducible randomness.*

Von Neumann understood well that it is difficult, if not outright impossible in general, to check empirically the randomness for individual systems, say for electrons or photons. In particular, he proceeded with the *statistical interpretation* of probability based on the mathematical model of von Mises [14,15] based upon *relative frequencies* after *admissible place selections.*

At the same time, it is just and fair to note that the aforementioned tendencies to ground physics, and by reductionism, all of science, in ontological indeterminism, have been strongly contested and fiercely denied by eminent physicists; most prominently by Einstein. Planck [16] (p. 539) (see also Earman [17] (p. 1372)) believed that causality could be neither generally proved nor generally disproved. He suggested to postulate causality as a working hypothesis, a heuristic principle, a sign-post (and for Planck the most valuable sign-post we possess) *“to guide us in the motley confusion of events”*.

This is a good place to remark that random features of an individual system can be discussed in the framework of *subjective probability theory.* The individual (irreducible) interpretation of quantum randomness due to von Neumann matches well with the subjective probability interpretation of quantum mechanics (QBism, see, e.g., [18,19]).

The main reason for keeping the statistical interpretation was that the aforementioned individual randomness of quantum systems was considered by von Neumann as one of the basic features of nature (and not of the human mind!). Von Neumann was sure that such a natural phenomenon must be treated statistically (by the same reason Bohr also treated quantum randomness statistically, see [20] for details).

In particular, von Neumann remarked [13] (pp. 301–302), that, for measurement of some quantity *R* for an ensemble of systems (of any origin),

*It is not surprising that R does not have a sharp value …, and that a positive dispersion exists. However, two different reasons for this behavior a priori conceivable:*

*1*. The individual systems S1,…,SN of our ensemble can be in different states, so that the ensemble [S1,…,SN] is defined by their relative frequencies. The fact that we do not obtain sharp values for the physical quantities in this case is caused by our lack of information: we do not know in which state we are measuring, and therefore we cannot predict the results.
*2*. All individual systems S1,…,SN are in the same state, but the laws of nature are not causal. Then, the cause of the dispersion is not our lack of information, but nature itself, which has disregarded the principle of sufficient cause.

These are characterizations of epistemic and ontic indeterminism, respectively. Von Neumann favored the second, ontic, case which he considered “important and new” (and which he believed to be able to corroborate [21]). Therefore, for von Neumann, quantum randomness is essentially a statistical exhibition of violation of causality, a violation of the principle of sufficient cause.

We compare this kind of randomness with classical interpretations of randomness, see, e.g., Chapter 2 [22]:

1. unpredictability (von Mises),
2. complexity-incompressibility (Kolmogorov, Solomonof, Chaitin),
3. typicality (Martin-Löf).

It seems that the interpretation of randomness as unpredictability (von Mises) is very close to the interpretation of quantum randomness as an exhibition of acausality.

The article by Pavicic and Megill [23], *Vector Generation of Quantum Contextual Sets in Even Dimensional Hilbert Spaces*, is a novel contribution to quantum contextuality theory. As is well known, the most elaborated contextual sets, which offer blueprints for contextual experiments and computational gates, are the Kochen–Specker sets. In this paper, a method of vector generation that supersedes previous methods is presented. It is implemented by means of algorithms and programs that generate hypergraphs embodying the Kochen-Specker property and that are designed to be carried out on supercomputers.

Recent years were characterized by the tremendous development of quantum technology. Quantum random generators are among the most important outputs of this development. As is pointed out in the review by Martínez et al. [24], *Advanced Statistical Testing of Quantum Random Number Generators*, the natural laws of the microscopic realm provide a fairly simple method to generate non-deterministic sequences of random numbers, based on measurements of quantum states. In practice, however, the experimental devices on which quantum random number generators are based are often unable to pass some tests of randomness. In this review, two such tests are briefly discussed, the challenges that have to be encountered in experimental implementations are pointed out. Finally, the authors present a fairly simple method that successfully generates non-deterministic maximally random sequences.

The connection between quantum logic and quantum probability is highlighted by Dalla Chiara et al. [25] in the paper entitled *Probabilities and Epistemic Operations in the Logics of Quantum Computation.* The authors stress that quantum computation theory has inspired new forms of quantum logic, called quantum computational logics. In this article, they investigate the epistemic operation (which is informally used in a number of interesting quantum situations): the operation “being probabilistically informed”.

In the paper entitled *Enhancing Extractable Quantum Entropy in Vacuum-Based Quantum Random Number Generator*, Guo et al. [26] commit to enhancing quantum entropy content in the vacuum noise based quantum RNG. They have taken into account main factors in this proposal to establish the theoretical model of quantum entropy content, including the effects of classical noise, the optimum dynamical analog-digital convertor (ADC) range, the local gain and the electronic gain of the homodyne system.

The work by Enríquez et al. [27], *Entanglement of Three-Qubit Random Pure States*, is devoted to studying entanglement properties of generic three-qubit pure states. There are obtained the distributions of both the coefficients and the only phase in the five-term decomposition of Acín et al. for an ensemble of random pure states generated by the Haar measure on U(8). Furthermore, the authors analyze the probability distributions of two sets of polynomial invariants. One of these sets allows us to classify three-qubit pure states into four classes. Entanglement in each class is characterized using the minimal Renyi–Ingarden–Urbanik entropy. The numerical findings suggest some conjectures relating some of those invariants with entanglement properties to be ground in future analytical work.

In the article *New Entropic Inequalities and Hidden Correlations in Quantum Suprematism Picture of Qubit States*, Margarita A. Man’ko and Vladimir I. Man’ko [28] considered an analog of Bayes’ formula and the nonnegativity property of mutual information for systems with one random variable. For single-qubit states, they presented new entropic inequalities in the form of the subadditivity and condition corresponding to hidden correlations in quantum systems. Qubit states are represented in the quantum suprematism picture, where these states are identified with three probability distributions, describing the states of three classical coins, and illustrating the states by Triada of Malevich’s squares with areas satisfying the quantum constraints.

In the article by Plotnitsky [29], *“The Heisenberg Method”: Geometry, Algebra, and Probability in Quantum Theory*, quantum theory is reconsidered in terms of the following principle, which can be symbolically represented as QUANTUMNESS→PROBABILITY→ALGEBRA. The principle states that the quantumness of physical phenomena, that is, the specific character of physical phenomena known as quantum, implies that our predictions concerning them are irreducibly probabilistic, even in dealing with quantum phenomena resulting from the elementary individual quantum behavior (such as that of elementary particles), which in turn implies that our theories concerning these phenomena are fundamentally algebraic, in contrast to more geometrical classical or relativistic theories, although these theories, too, have an algebraic component to them.

The work by Delgado [30], *SU(2) Decomposition for the Quantum Information Dynamics in 2d-Partite Two-Level Quantum Systems*, presents a formalism to decompose the quantum information dynamics in $SU(2^{2d})$ for 2d-partite two-level systems into $2^{d-1}$SU(2) quantum subsystems. It generates an easier and more direct physical implementation of quantum processing developments for qubits.

The paper by Marius Nagy and Naya Nagy [31], *An Information-Theoretic Perspective on the Quantum Bit Commitment Impossibility Theorem*, proposes a different approach to pinpoint the causes for which an unconditionally secure quantum bit commitment protocol cannot be realized, beyond the technical details on which the proof of Mayers’ no-go theorem is constructed.

In the Copenhagen approach to quantum mechanics as characterized by Heisenberg, probabilities relate to the statistics of measurement outcomes on ensembles of systems and to individual measurement events via the actualization of quantum potentiality. In the review by Jaeger [32], *Developments in Quantum Probability and the Copenhagen Approach*, brief summaries are given of a series of key results of different sorts that have been obtained since the final elements of the Copenhagen interpretation were offered and it was explicitly named so by Heisenberg—in particular, results from the investigation of the behavior of quantum probability since that time, the mid-1950s. This review shows that these developments have increased the value to physics of notions characterizing the approach which were previously either less precise or mainly symbolic in character, including complementarity, indeterminism, and unsharpness.

A new way of orthogonalizing ensembles of vectors by “lifting” them to higher dimensions is introduced by Havlicek and Svozil [33] entitled *Dimensional Lifting through the Generalized Gram-Schmidt Process*. This method can potentially be utilized for solving quantum decision and computing problems.

Recently the mathematical formalism and methodology of quantum theory started to be widely applied outside of physics, especially in psychology, decision making, social and political science (see, e.g., [34]). This special issue contains one paper belonging to this area of research, the article of Khrennikov et al. [35], *On Interpretational Questions for Quantum-Like Modeling of Social Lasing*. The formalisms of quantum field theory and theory of open quantum systems are applied to modeling socio-political processes on the basis of the social laser model describing *stimulated amplification of social actions.* The main aim of this paper is establishing the socio-psychological interpretations of the quantum notions playing the basic role in lasing modeling.

The article by Paul Ballonoff [36], *Paths of Cultural Systems*, is also devoted to applications outside physics, namely to anthropology. A theory of cultural structures predicts the objects observed by anthropologists. A viable history (defined using pdqs) states how an individual in a population following such history may perform culturally allowed associations, which allows a viable history to continue to survive. The vector states on sets of viable histories identify demographic observables on descent sequences.

We hope that the reader will enjoy the present issue, which will be useful to experts working in all domains of quantum physics and quantum information theory, ranging from experimenters, to theoreticians and philosophers.

The cover of this electronic book was created by Renate Quehenberg and the editors would like to thank her for the graphical contribution to this special issue.

## References

[1] Khrennikov A., Weihs G. (2012). Preface of the special issue Quantum foundations: Theory and experiment. Found. Phys..

[2] Bengtsson I., Khrennikov A. (2011). Preface. Found. Phys..

[3] D’Ariano G.M., Jaeger G., Khrennikov A., Plotnitsky A. (2014). Preface of the special issue Quantum theory: Advances and problems. Phys. Scr..

[4] Khrennikov A., de Raedt H., Plotnitsky A., Polyakov S. (2015). Preface of the special issue Probing the limits of quantum mechanics: Theory and experiment, Volume 1. Found. Phys..

[5] Khrennikov A., de Raedt H., Plotnitsky A., Polyakov S. (2015). Preface of the special issue Probing the limits of quantum mechanics: Theory and experiment, Volume 2. Found. Phys..

[6] Von Schweidler E. (1906). Über Schwankungen der Radioaktiven Umwandlung.

[7] Hiebert E.N. (2000). Common frontiers of the exact sciences and the humanities. Phys. Perspect..

[8] Stöltzner M. (1999). Vienna indeterminism: Mach, Boltzmann, Exner. Synthese.

[9] Exner F.S. (2016). Über Gesetze in Naturwissenschaft und Humanistik: Inaugurationsrede Gehalten am 15. Oktober 1908.

[10] Exner F.S. (1922). Vorlesungen über die Physikalischen Grundlagen der Naturwissenschaften.

[11] Schrödinger E. (1929). Was ist ein Naturgesetz?. Naturwissenschaften.

[12] Born M. (1926). Zur Quantenmechanik der Stoßvorgänge. Z. Phys..

[13] Von Neuman J. (1955). Mathematical Foundations of Quantum Mechanics.

[14] Von Mises R. (1919). Grundlagen der Wahrscheinlichkeitsrechnung. Math. Z..

[15] Von Mises R. (1964). The Mathematical Theory of Probability and Statistics.

[16] Planck M. (1932). The concept of causality. Proc. Phys. Soc..

[17] Earman J., Butterfield J., Earman J. (2007). Aspects of determinism in modern physics. Part B: Philosophy of Physics, Handbook of the Philosophy of Science.

[18] Fuchs C.A. (2002). Quantum mechanics as quantum information (and only a little more). Quantum Theory: Reconsideration of Foundations.

[19] Fuchs C.A., Schack R. (2014). QBism and the Greeks: why a quantum state does not represent an element of physical reality. Phys. Scr..

[20] Plotnitsky A., Khrennikov A. (2015). Reality without realism: On the ontological and epistemological architecture of quantum mechanics. Found. Phys..

[21] Dieks D. (2017). Von Neumann’s impossibility proof: Mathematics in the service of rhetorics. Stud. Hist. Philos. Mod. Phys..

[22] Khrennikov A. (2016). Probability and Randomness: Quantum Versus Classical.

[23] Pavicic M., Megill N.D. (2018). Vector Generation of Quantum Contextual Sets in Even Dimensional Hilbert Spaces. Entropy.

[24] Martínez A.C., Solis A., Rojas R.D.H., U’ Ren A.B., Hirsch J.G., Castillo I.P. (2018). Advanced Statistical Testing of Quantum Random Number Generators. Entropy.

[25] Dalla Chiara M.L., Freytes H., Giuntini R., Leporini R., Sergioli G. (2018). Probabilities and Epistemic Operations in the Logics of Quantum Computation. Entropy.

[26] Guo X., Liu R., Li P., Cheng C., Wu M., Guo Y. (2018). Enhancing Extractable Quantum Entropy in Vacuum-Based Quantum Random Number Generator. Entropy.

[27] Enríquez M., Delgado F., Zyczkowski K. (2018). Entanglement of Three-Qubit Random Pure States. Entropy.

[28] Man’ko M.A., Man’ko V.I. (2018). New Entropic Inequalities and Hidden Correlations in Quantum Suprematism Picture of Qubit States. Entropy.

[29] Plotnitsky A. (2018). The Heisenberg Method: Geometry, Algebra, and Probability in Quantum Theory. Entropy.

[30] Delgado F. (2018). *S**U*(2) Decomposition for the Quantum Information Dynamics in 2d-Partite Two-Level Quantum Systems. Entropy.

[31] Nagy M., Nagy N. (2018). An Information-Theoretic Perspective on the Quantum Bit Commitment Impossibility Theorem. Entropy.

[32] Jaeger G. (2018). Developments in Quantum Probability and the Copenhagen Approach. Entropy.

[33] Havlicek H., Karl Svozil K. (2018). Dimensional Lifting through the Generalized Gram-Schmidt Process. Entropy.

[34] Khrennikov A. (2010). Ubiquitous Quantum Structure: From Psychology to Finances.

[35] Khrennikov A., Alodjants A., Trofimova A., Tsarev D. (2018). On Interpretational Questions for Quantum-Like Modeling of Social Lasing. Entropy.

[36] Ballonoff P. (2018). Paths of Cultural Systems. Entropy.

